# The Role of Selected Factors in the Development and Consequences of Alcohol Dependence

**Published:** 2008

**Authors:** Rebecca Gilbertson, Robert Prather, Sara Jo Nixon

**Keywords:** Alcoholism, alcohol and other drug (AOD) dependence, risk factors, genetic factors, family factors, environmental factors, gender differences, family AOD use (AODU) history, comorbidity, multiple drug use, age of AODU onset, AOD effects, brain

## Abstract

Gender, family history, comorbid psychiatric and substance use disorders, and age all influence a person’s risk for alcoholism. In addition, these factors interact with alcoholism to influence neurocognitive functioning following detoxification. This article examines these factors and considers how they interact with each other. This complexity reinforces the need for both animal and human studies and suggests multiple factors that may be sensitive to differential prevention, intervention, and treatment efforts. Thus, it is imperative that hypothesis-driven research designs be directed to identifying the relative potency of these factors and their interactions.

Many risk factors contribute to both the development of alcohol dependence and its long-term consequences. This complexity no doubt contributes to the heterogeneity in research findings, complicating treatment as well as identifying multiple avenues for intervention efforts. A comprehensive review of the risk factors for alcoholism is beyond the scope of this article. Rather, the following sections will focus on five major risk factors: gender, family history, psychiatric comorbidity, comorbid substance abuse, and age. In addition to discussing how these factors influence alcoholism risk, the article also will examine how they interact with alcoholism to influence neurocognitive functioning following detoxification. Thus, information in this article focusing on neurocognitive performance in alcoholics generally refers to data obtained from people who are recently sober, beyond the stage of detoxification, and not currently on medication that might affect neurocognitive function. Although this condition restrains the generalizability of the results, it provides a more considered review of the neurocognitive impact of alcohol dependence.

Before proceeding, it is necessary to clarify and define terms used in this article. Throughout the past two decades, the clinical definition of “alcoholic” and “alcoholism” has evolved, as evidenced in classification issues detailed in the *Diagnostic and Statistical Manual of Mental Disorders* (DSM) used by mental health professionals (American Psychiatric Association [APA] 1980, 1987, 1994). For example, as programmatic research was being broadly initiated in the 1970s and 1980s, research inclusion criteria often did not differentiate between alcohol abuse and dependence. Thus, studies often included participants with either disorder in a single group referred to as those with “an alcohol use disorder” or “alcoholism.” As the complexity of alcohol use disorders (AUDs) was better appreciated and clinically and scientifically meaningful distinctions between alcohol-related diagnoses were made, groups within studies became more strictly defined. Although it is not universally accepted, the term “alcoholic” now is generally applied within addiction research to people with a DSM–IV “alcohol dependence” diagnosis. Given this shift in perspective, it is important to recognize that earlier studies (e.g., those including data from the 1970s, 1980s, and early 1990s) may include people with either or both diagnoses.

## Gender

### Gender As a Risk Factor for Alcohol Dependence

Researchers have investigated drinking behaviors, their etiology, and outcomes among women for several decades (e.g., Fabian et al. 1984; Glenn and Parsons 1992; Nixon and Glenn 1995; Sullivan et al. 2002). These data suggest that the rate of progression of problematic drinking and subsequent risk for alcohol-related consequences may be different for men and women. Historically, men have reported an earlier age of onset of alcohol use initiation than women (Chou and Dawson 1994; Gomberg 1993). One large national study (Project MATCH) (Randall et al. 1999) of people seeking treatment for alcoholism supported these findings and further showed that men displayed evidence of problematic drinking behaviors (i.e., regular intoxication, loss of control over drinking) earlier than women.

**Figure 1:**
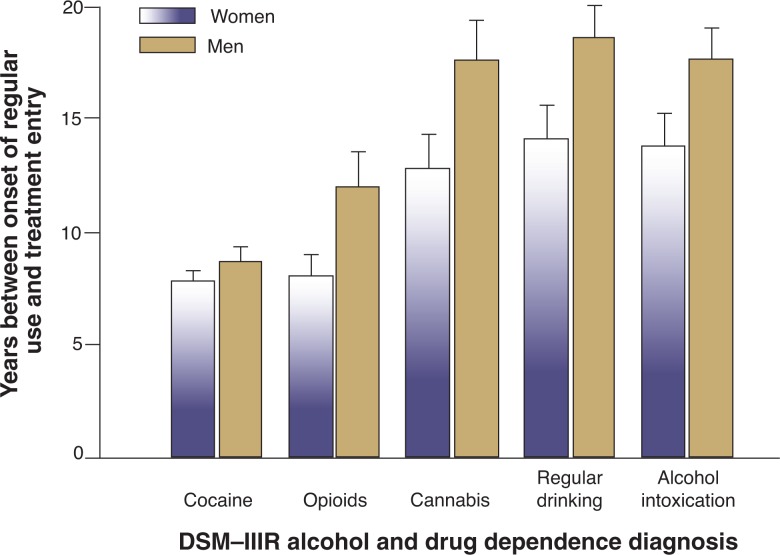
Gender telescoping. Bars represent number of years (means ± standard error of the mean) elapsed between onset of regular substance use and entry into substance abuse treatment by gender and substance dependence diagnosis. Women experienced fewer pretreatment years of regular use of opioids (*P* = 0.03), cannabis (*P* = 0.01), regular alcohol drinking (*P* = 0.03), and regular alcohol intoxication (*P* = 0.09) than men (Hernandez-Avila et al. 2004). SOURCE: Used with permission. 2004 Elsevier Ireland Ltd. Figure 1, p. 368. Hernandez-Avila, C.A.; Rounsaville, B.J.; and Kranzler, H.R. Opioid-, cannabis- and alcohol-dependent women show more rapid progression to substance abuse treatment. *Drug and Alcohol Dependence* 74(3):265–272, 2004. PMID: 15194204

However, not all studies support gender differences in age of onset of regular alcohol use, and some suggest that age of initial use may be increasingly similar for both genders, at least for those who ultimately seek treatment. For example, Hernandez-Avila and colleagues (2004) found remarkable similarity in age of onset of regular use between male and female substance abusers, reporting no significant differences between men and women with current alcohol dependence diagnoses with regard to age of onset of regular drinking or age of onset of regular alcohol intoxication. They did, however, find that women progressed from regular use to treatment more quickly than men (see figure 1). This latter finding is consistent with other data demonstrating that women progress through the stages of regular intoxication, drinking problems, and loss of control over drinking more quickly than men. That is, women demonstrate a “telescoping” of disease progression and experience more severe consequences more quickly (Diehl et al. 2007; Hernandez-Avila et al. 2004; Mann et al. 2005; Randall et al. 1999).

The telescoping effect in alcoholic women may be associated with several factors. First, the immediate personal and professional costs to women may be greater, as suggested by data indicating that women report more psychiatric, medical, and employment consequences from heavy drinking compared with men (Hernandez-Avila et al. 2004). Second, telescoping may be related to gender differences in physiology. For example, among men and women consuming similar amounts of alcohol (per body weight) (Mann et al. 2005), women may experience higher blood alcohol concentrations because of metabolic differences (see Ammon et al. 1996; Baraona et al. 2001; Frezza et al. 1990). Thus, certain complications that may contribute to the telescoping effect in alcoholic women could be attributed to achieving and sustaining higher blood alcohol levels than alcoholic men when equivalent doses of alcohol are consumed.

### Gender As a Factor in Alcohol’s Effects on the Brain

The effects of telescoping on the brain and cognition in alcoholic women remain unclear (Hommer et al. 2001, 2003; Pfefferbaum et al. 2001). Alcoholic men and women in recovery often display similar neuropsychological deficits, although as discussed, women have a shorter course of disease (see Fabian et al. 1984; Flannery et al. 2007; Glenn and Parsons 1992; Mann et al. 2005; Nixon and Glenn 1995; Randall et al. 1999). Thus, the female brain may be differentially sensitive to the neurotoxic effects of alcohol (Hommer et al. 2001; Hommer 2003).

Neuroimaging techniques have allowed further investigation of the macrostructural (i.e., the size or volume of a brain structure) and microstructural (i.e., the small constituents of white matter, such as myelin) integrity of white matter tracts within the brains of alcoholic men and women (Pfefferbaum et al. 2002, 2006; Pfefferbaum and Sullivan 2002). Although alcoholic men were observed to have macrostructural aberrations (including smaller volume) in the pons, corpus callosum, and cortical white matter, alcoholic women did not display such differences (Pfefferbaum et al. 2002). However, the microstructural integrity of cortical and callosal white matter was affected to similar extents in both alcoholic men and women, even though the alcoholic women had drunk far less alcohol in their lifetimes than the men (Pfefferbaum and Sullivan 2002). These results suggest that white matter areas within the brain are affected by alcohol dependence; however, they may be affected differently in women compared with men. Further, although certain areas may not show overt volume differences, alcohol dependence still may affect the microstructural integrity and potentially compromise brain function. Research is ongoing regarding the microstructural integrity of the brain following alcohol dependence, with results suggesting the involvement of multiple brain regions (Pfefferbaum et al. 2006).

Despite the strength of these findings, it should again be noted that much of the work conducted with women has not fully accounted for metabolic (pharmacokinetic) differences between the genders. These differences result in greater alcohol exposure of liver and brain tissue in women as opposed to men, even when an equivalent dose of alcohol is consumed (Baraona et al. 2001; Dettling et al. 2007; Frezza et al. 1990; Hommer et al. 2001). Thus, women may not be differentially sensitive to alcohol, per se, but rather may be chronically exposed to higher blood alcohol levels even at lower doses.

## Family History

### Family History As a Risk Factor for Alcohol Dependence

It is well established that alcoholism runs in families. Furthermore, adoption studies, family pedigree studies, and twin studies consistently support the role of genetic risk rather than familial transmission for alcohol dependence (Carlson et al. 2002; Cloninger et al. 1981; Cotton 1979; McGue 1997; Russell 1990). Estimates vary, but it generally is accepted that offspring of alcoholics are approximately four times more likely to develop alcoholism than people without such a history (Russell 1990), even if they are not reared with an alcoholic parent. Most early research studied male offspring of male alcoholics. This limitation led to the early conclusion that men were more likely to experience the familial form of the disease, whereas women were more likely to experience a reactive form associated with psychiatric comorbidity, empty-nest syndrome, or related factors.

**Figure 2:**
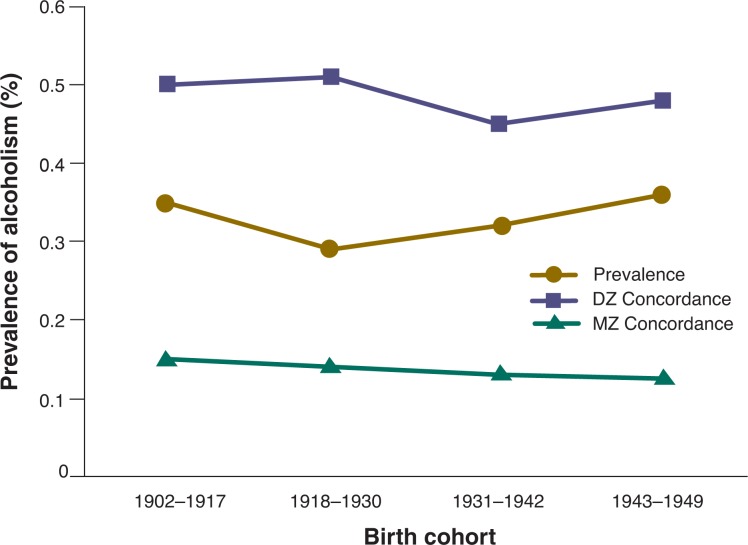
Concordances for monozygotic and dizygotic twins. Prevalence of alcoholism in twins and twin concordance of monozygotic (MZ) and dizygotic (DZ) twins for alcoholism in a study of 8,935 pairs of Swedish male twins. Adapted from data reported by Kendler et al. (1992). Squares demarcate 1 standard error (McGue 1999). SOURCE: Derived from: 1999 American Psychological Society, figure 1, p. 110. McGue, M. The behavioral genetics of alcoholism. *Current Directions in Psychological Science* 8(4):109–115, 1999.

With continued and more broadly developed research, these assumptions have been modified. Widely cited studies using male and female monozygotic and dizygotic twins suggest that genes, environment, and their interaction are potent contributors to the development of alcohol dependence in both genders (Heath et al. 1997; Krueger et al. 2002; McGue 1997, 1999; Prescott and Kendler 1999; Sigvardsson et al. 1996) (see figure 2). Approximately 40 percent of the variance for alcoholism onset in men (Prescott and Kendler 1999) and 60 percent of this variance in women can be attributed to genes (Kendler et al. 1992).

Many twin studies considered paternal alcoholism rather than both paternal and maternal input (Kendler et al. 1992). Attention to direct maternal contribution has been limited for numerous reasons. One of the predominant reasons is that the study of maternal genetic impact on alcoholism risk was restrained by the concern that offspring would be more likely to be exposed to alcohol in utero, and, thus, results regarding genetic risk would be confounded with the effects of early exposure (see Streissguth and O’Malley 2000). However, Hill and colleagues (e.g., Hill and Steinhauer 1993; Hill et al. 1995), controlling for prenatal exposures, demonstrated that daughters of alcoholic mothers also were at increased risk for alcoholism, even without paternal alcoholism.

The Collaborative Study on the Genetics of Alcoholism (COGA), in conjunction with other studies, has implicated several genetic markers in which variations appear to increase risk for alcohol dependence and related disorders. These include genes associated with the acetylcholine receptor, the receptor for the major inhibitory neurotransmitter, γ-aminobutyric acid (GABA), and those associated with alcohol metabolism (see Edenberg and Foroud 2006; Porjesz and Rangaswamy 2007). Edenberg and Faroud (2006) also reported preliminary data on several other loci, one of which is associated with the bitter taste receptor. Agrawal and colleagues (2008) extended work with the COGA sample and further expanded the list of potential genes by implicating regions of chromosomes believed to affect neurophysiology in complex ways, including signal transduction across cell membranes within the brain. This group also has implicated the role of signal transduction in modulating risk in an additional study (Dick et al. 2007).

If, as noted above, an estimated 40 to 60 percent of the risk for alcoholism can be attributed to genetic factors, a sizable remaining variance is associated with environmental factors and gene-by-environment interactions. Finnish and Canadian twin studies (Jang et al. 2000, 2001; Kaprio et al. 2002) indicate that environmental factors such as geographical locations with high consumption rates, religiosity/moral views, and exposure to antisocial personality traits may interact with genetics to increase risk for alcohol dependence. Although some studies suggest that exposure to paternal alcoholism during childhood does not seem to contribute to greater risk for alcoholism later in life (Duncan et al. 2006), other studies show that a low-risk environment (i.e., absence of paternal alcoholism) can reduce the risk of developing alcoholism later in life even in people with greater family density of alcoholism (Jacob et al. 2003). Thus, both environmental and genetic factors influence risk for alcohol dependence and related disorders.

### Family History As a Factor in Alcohol’s Effects on the Brain

Not only does a positive family history increase the risk for developing alcoholism, it also may influence neurocognitive functioning among people who have such a history but are not themselves alcoholic. For example, several studies have examined mental processes in offspring (primarily sons) of male alcoholics (Giancola et al. 1996; Tarter et al. 2003). These studies have observed subtle, yet significant, deficits among family history positive (FH^+^) participants, particularly on tasks such as problem solving and abstraction, often referred to as executive cognitive functioning (ECF) (Aytaclar et al. 1999; Giancola et al. 1996; Tarter 2002).

Other studies have examined neurophysiological functioning in FH^+^ nonalcoholics. Many of these studies have used noninvasive brain electro-physiology to measure the brain’s electrical responses with electrodes placed on the scalp. These studies suggest aberrations in the neurophysiology underlying target detection, memory updating, and working memory in both male and female off-spring of alcoholics (Begleiter et al. 1984; Carlson et al. 2004; Hill et al. 1995; Rangaswamy et al. 2007). Importantly, however, such aberrations are not uniformly observed, and researchers have documented eventual normalization of these responses in subgroups. Thus, it appears that although some FH^+^ individuals may demonstrate long-lived, yet subtle, deficits in these measures; for others, these deficits suggest a development lag in fundamental brain processes (Bauer and Hesselbrock 1999; Hill et al. 1999; Hill and Shen 2002).

Additional studies have used neuroimaging procedures such as magnetic resonance imaging (MRI) or related procedures to examine brain function in FH^+^ individuals (Hill et al. 2007; McNamee et al. 2008). These studies also have reported brain changes in FH^+^ adolescents, showing decreased activation in the frontal region of the brain (an area typically associated with ECF) as well as areas of the brain associated with social cognition and empathy. Additionally, brain response to alcohol cues may differ between FH^+^ and FH^−^ individuals. Bartholow and colleagues (2007) found that FH^+^ individuals had greater P300^1^ amplitude response to alcohol cues versus nonalcohol cues.

It remains unclear the extent to which these aberrations or alterations in brain function serve as markers for risk for developing alcohol dependence or whether they reflect more general behavior patterns associated with disorders that commonly co-occur with alcohol dependence, such as childhood behavior disorders or other externalizing disorders.

**Figure 3:**
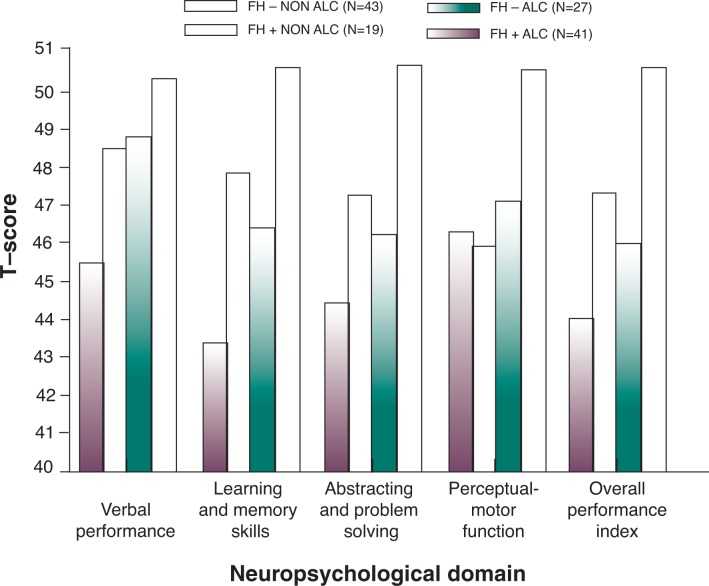
Role of family history in cognition. Mean performances on the cognitive neuropsychological test clusters for family history–positive (FH^+^) and family history–negative (FH^−^) alcoholics and nonalcoholic peer control subjects. Standard scores were based on the nonalcoholic FH^−^ group. The FH^+^ alcoholics differed significantly from the FH^−^ control subjects on all of the performance clusters. The FH^−^ alcoholics and FH^+^ control subjects did not differ significantly from each other on any of the clusters. SOURCE: Derived from Parsons 1989.

Because (1) the majority of chronic alcohol studies are conducted using treatment-seeking alcoholics and (2) the large majority of treatment-seeking alcoholics have positive family histories, there has been some question to whether neurocognitive deficits (including behavioral and neuroimaging aberrations) are associated with FH^+^ status rather than alcohol dependence, per se. Because so few studied alcoholics are FH^−^, it is difficult to make statistically sound comparisons. Parsons and colleagues (see for example Glenn and Parsons 1989; Parsons 1994) conducted a series of studies explicitly examining this hypothesis. The studies compared FH^+^ and FH^−^ detoxified alcoholics without confounding psychiatric (e.g., schizophrenia or bipolar disorders) or medical (e.g., epilepsy or history of head injury) disorders on a battery of neuropsychological tests. Consistent with the hypothesis that chronic excessive alcohol use is neurotoxic, alcoholic participants demonstrated deficits that could not be accounted for by FH (see figure 3). Interestingly, a recent study (Fein and Chang 2008) considering the role of FH found that density of FH, rather than alcohol use, was negatively associated with impaired decisionmaking ability in alcoholics. Specifically, alcoholics with a greater density of affected family members showed decrements in how the brain responds to negative consequences of behavior when compared with alcoholics lacking such histories (Fein and Chang 2008).

## Psychiatric Comorbidity

### Psychiatric Comorbidity As a Risk Factor for Alcohol Dependence

People with AUDs frequently meet criteria for other psychiatric disorders as well. For example, early data gathered through the National Institute of Mental Health Epidemiological Catchment Area Project revealed significant levels of comorbidity, with 3.8 percent of those with a lifetime diagnosis of alcohol dependence also meeting criteria for a lifetime diagnosis for a major psychotic disorder (Regier et al. 1990). More recent data from the National Epidemiologic Survey on Alcohol and Related Conditions (NESARC) reveal that among individuals with alcohol dependence, 15.15 percent and 17.75 percent also met criteria for a depressive disorder or anxiety diagnosis, respectively (Grant et al. 2004). Personality disorders also are common among alcoholics. For example, alcoholics are reportedly 21 times more likely to have a diagnosis of antisocial personality disorder (ASPD) than are nonalcoholics (Reiger et al. 1990). Further, people with ASPD appear to be at greater risk for severe AUDs (i.e., more criteria for lifetime abuse and dependence met, greater frequency of heavy-drinking days) compared with people with a conduct disorder diagnosis without ASPD or those who met criteria for ASPD without conduct disorder prior to age 15 (Goldstein et al. 2007). Interestingly, these authors conclude that the relationship between ASPD and AUDs is similar for men and women.

Given these rates of comorbidity, it is important to consider the extent to which AUDs may be causally related to other diagnoses. For example, do people drink because they are depressed or are they depressed because they drink? Similarly, do people with social anxiety and alcohol problems reduce drinking when the anxiety is treated? Despite this entanglement of AUDs with other psychiatric disorders, it is evident that the development of alcohol dependence is not contingent on the presence of another psychiatric disorder. That is, alcohol dependence may develop in individuals without other disorders, and individuals with other diagnoses do not necessarily develop AUDs. However, common genetic and environmental factors, as well as gene-by-environment interaction, may place individuals with alcohol disorders at a higher risk for psychiatric disorders compared with those without such comorbidities. Thomas and colleagues (2008) recently addressed this complexity. They reported that among patients with both social anxiety and alcohol use problems, pharmacological treatment of social anxiety resulted in reduced anxiety symptoms but did not reduce drinking. However, it did reduce the percentage of times that study participants reported drinking to reduce anxiety. Thus, at least for this sample, there was a dissociation between levels of social anxiety and alcohol consumption, even among those who believed they used alcohol to “reduce social fears.”

In summary, there is high comorbidity between AUDs and other psychiatric disorders. Determining to what extent the onset of one precedes or follows another is complicated by overlapping symptomatology, individual differences in symptom onset, and methods of reporting. From a clinical perspective, it is clear that regardless of order of onset, multiple disorders must be treated individually and cooperatively (McGovern and McLellan 2008). Treating only one of the disorders is unlikely to produce effective psychiatric recovery (Grant et al. 2004).

### Psychiatric Comorbidity As a Factor in Alcohol’s Effect on the Brain

There is a rich literature considering the interaction of alcoholism and other major psychiatric disorders on neurocognitive function (Glenn et al. 1993; Maurage et al. 2008; Thoma et al. 2007; Uekermann et al. 2003). In an older, yet methodologically interesting, study, Nixon and colleagues (1996) examined a limited number of cognitive processes in dually diagnosed schizophrenic inpatients. In contrast to many cross-sectional studies, they were able to recruit four study groups: three groups of inpatients (schizophrenics, those with AUDs, and those with both a schizophrenia and an AUD diagnosis) as well as a group of community control subjects. Consistent with the heterogeneity in the field, control subjects were generally, although not always, significantly superior to the other groups. Of more immediate interest was the finding that dually diagnosed schizophrenics were not more impaired than schizophrenics without an AUD. Although this result is somewhat counterintuitive, it is consistent with other studies suggesting that schizophrenics who develop substance use disorders (excluding nicotine) may have improved interpersonal skills relative to their non-addicted cohorts (Dixon et al. 1991).

Comorbid personality disorders have been systematically examined less frequently, with the exception of ASPD (Bauer and Hesselbrock 1999; Ceballos et al. 2003; Costa et al. 2000; Stevens et al. 2001). Some researchers have argued that much of the presumed alcohol-related cognitive compromise actually is attributable to underlying ASPD. This perspective may be particularly relevant when dependent variables associated with behavioral inhibition and impulse control are considered. However, these types of variables are not the only ones impacted by ASPD status. Ceballos and colleagues (2003) examined semantic processing ability in alcoholics and nonalcoholics with and without ASPD. Regression analyses showed that being alcoholic and having ASPD resulted in poorer semantic processing compared with control subjects. These results suggest that although alcohol dependence and ASPD are frequently comorbid, neurocognitive changes seen in recently sober alcoholics cannot be accounted for by ASPD status alone.

Brain function is affected by both alcohol dependence and other psychiatric disorders. However, when the influence of comorbid conditions is accounted for, most studies reveal changes in brain structure and function associated with alcohol dependence, separate from other disorders. As neuroimaging techniques become increasingly sensitive, specific influences on particular brain systems, especially within white matter connections, may be more evident.

## Comorbid Substance Use

### Comorbid Substance Use As a Risk Factor in Alcohol Dependence

The co-occurrence of alcohol and other drug use disorders is well recognized. Data analyzed from the National Comorbidity Survey revealed that 29.5 percent of men and 34.7 percent of women who met criteria for alcohol dependence also were drug dependent (Kessler et al. 1997). Importantly, AUDs were found to precede drug problems in 25.6 percent of men and 20.0 percent of women (Kessler et al. 1997). NESARC data reveal a positive and significant relationship between current alcohol use and specific drug disorders such as cocaine dependence (Stinson et al. 2005), suggesting that alcohol use increased the risk for other drug use disorders. Other studies that used NESARC data and controlled for sociodemographic characteristics found that people with alcohol dependence were almost 19 times more likely than people without alcohol dependence to meet criteria for drug dependence in the last 12 months. When controlling for comorbid psychiatric disorders, people with alcohol dependence were 7.5 times more likely than others to have a drug dependence diagnosis (Hasin et al. 2007).

**Figure 4:**
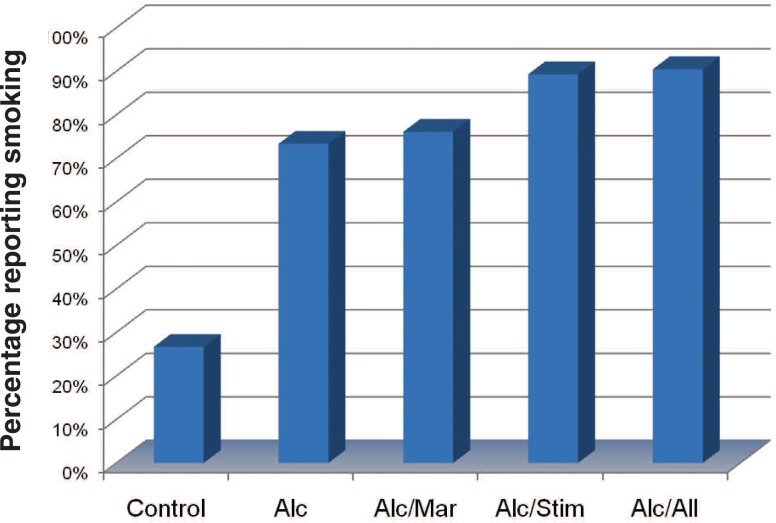
Prevalence of smoking in treatment-seeking substance-abusing subgroups. Alc = alcohol use disorder; Alc/All = alcohol and any drug use disorder; Alc/Mar = alcohol and marijuana use disorders; Alc/Stim = alcohol and stimulant use disorders; Control = community comparison group with no psychiatric disorder and no substance use disorder. SOURCE: Ceballos, NA. Tobacco use, alcohol dependence, and cognitive performance. *Journal of General Psychology* 133(4):375–388, 2006. PMID:17128957

Nicotine use disorder, demonstrated primarily through tobacco cigarette smoking, also commonly co-occurs with AUDs. People with nicotine use disorder are two to three times more likely to be diagnosed with AUDs, and a current diagnosis of either increases risk for being diagnosed with the other in the future (Grucza and Bierut 2006; Sher et al. 1996). The rate of tobacco use among treatment-seeking alcoholics and other substance abusers is roughly three times that of the general population with rates ranging from 76 percent to more than 90 percent (Collins and Marks 1995; DiFranza and Guerrera 1990). Ceballos and colleagues (2006) reported similar prevalence data (see figure 4). Research with adolescents suggests that alcohol, drug (i.e., marijuana), and smoking behaviors frequently develop around the same time (Faeh et al. 2006).

Data from the COGA project showed that variations in certain genetic factors may contribute to risk for a particularly severe form of alcohol dependence and comorbid drug dependence (Dick et al. 2007). People with alcohol dependence and comorbid drug dependence displayed earlier age of onset of regular drinking, higher rates of ASPD, conduct disorder, and novelty seeking. Thus, Dick and colleagues (2007) suggested that the gene variant may be linked to behavioral disinhibition traits rather than drug use, per se. This type of association is consistent with findings reported by Faeh and colleagues (2006). Of particular clinical relevance are recent findings revealing that people with comorbid alcohol and other drug disorders are more likely to seek treatment than those with an alcohol disorder alone (Stinson et al. 2005).

### Comorbid Substance Use Disorders As a Factor in Alcohol’s Effects on the Brain

The strong association between alcohol and tobacco use may be mediated through several variables, including the activation of underlying brain reward systems. In addition, the cognitive enhancing effect of acute nicotine may contribute to the high levels of comorbidity. Whereas alcohol dependence is associated with subtle, yet significant, cognitive dysfunction, acute nicotine is known to enhance cognition, particularly processes associated with vigilance and attentional aspects of working memory (Heishman 1998; Rodway et al. 2000). Given the opposing effects, it follows that acute nicotine may serve to compensate for deficits associated with alcohol dependence. If so, the strong association between the use of the two substances may not lie entirely in the reward systems or shared genetic risks but also in their functional interaction. Recent data revealing that alcoholics are differentially sensitive to acute nicotine compared with community smoking control subjects are consistent with this conclusion (Nixon et al. 2007). These interactions have significant implications for the use of aggressive nicotine replacement therapy, particularly in the early stages of recovery when cognitive processes may be most compromised.

The effects of chronic smoking (chronic nicotine use) on brain structure and function also have been studied in alcoholics. These findings suggest that chronic smoking alcoholics show decrements in neurocognitive functioning and anatomical brain structure as compared to nonsmoking alcoholics (Durazzo et al. 2007). Further, these differences persist through recovery from alcoholism (Durazzo et al. 2006). Thus, although acute nicotine administration improved neurocognitive function in alcoholics (e.g., Nixon et al. 2007), chronic cigarette smoking is associated with decrements in brain structure and function. These data suggest that smoking effects may be quite different than the effects of nicotine itself.

## Age

### Age As a Risk Factor in Alcohol Dependence

Much of the literature concerning age as a risk for alcoholism has focused on defining two subtypes of alcoholics, those who were dependent on alcohol at an early age (before age 25) (Gilman et al. 2007; Glenn and Nixon 1991, 1996; Roache et al. 2008) and those who develop alcoholism later in life (Atkinson 2002; Atkinson et al. 2003). Early work suggested that early-onset alcoholics were more likely than late-onset alcoholics to have job problems, to be younger when they first drank alcohol, to have a higher rate of maternal alcoholism, and to have childhood behavioral disorders and antisocial behaviors (Glenn and Nixon 1991, 1996). Recent data further reinforce the importance of early age of drinking by demonstrating that people who have their first drink prior to age 15 are more likely than others to develop an AUD (Dawson et al. 2008).

In contrast, those who develop alcohol problems after age 60 are characterized by having more biomedical versus psychosocial consequences, compared with early-onset alcoholics, and are more likely to have alcohol–medication interactions (Atkinson 2002). Further, later-onset alcoholics are likely to have a history of heavy alcohol use and meet dependence criteria attributed to life stress or psychiatric comorbidity (Atkinson 2002; Brennan et al. 1999; Schutte et al. 1998). NESARC data reveal that the prevalence of alcoholism in older individuals may be increasing, possibly following general population trends (Hasin et al. 2007; National Projections Program, U.S. Bureau of the Census 2008.)

### Age As a Factor in Alcohol’s Effects on the Brain

The potential interaction of chronic alcoholism and brain aging, also referred to as the premature aging hypothesis, has been a long-standing research interest. One version of the hypothesis suggested that chronic alcoholism prematurely aged the brains of young adults. Findings from early studies suggesting that brain structure and function in young alcoholics resembled older normal control subjects were consistent with this conclusion (Blusewicz et al. 1977; Holden et al. 1988; Graff-Radford et al. 1982). Most of these studies, however, used cross-sectional designs. Although some alcohol-related brain changes were similar to those caused by aging, age-appropriate control subjects were not always included, and thus it was difficult to conclude that the differences were not the result of factors other than alcohol exposure. Continuing research regarding this important question generally suggests that alcoholism, per se, does not cause premature aging in younger drinkers (Oscar-Berman 2000; Oscar-Berman and Marinkovi 2007).

This conclusion does not eliminate an interaction of alcohol and aging. The alternative version of the premature aging hypothesis suggests that older drinkers may be more sensitive to the neurotoxic effects of alcohol than younger drinkers. This hypothesis has been supported by a number of studies that accounted for quantity and frequency of use as well as drinking occasions and number of acute withdrawals (Oscar-Berman and Marinkovi 2003). That is, even when alcohol exposure, per se, can be statistically controlled for, older alcoholics show greater effects. This susceptibility has been particularly evident in structural brain-imaging studies (see the article by Rosenbloom and Pfefferbaum in this issue, pp. 362–376) (e.g., Pfefferbaum et al. 1992, 1996, 1997) and more specifically with analysis of white matter microstructural integrity (Pfefferbaum et al. 2006). Some studies also have suggested gender-by-age interactions. For example, men showed significant associations between age and decrements in prefrontal and entire cortical gray matter, sulcal volume, and third ventricular volume (Pfefferbaum et al. 1997, 2001), whereas the association between ventricular expansion and advancing age were prominent in alcoholic women.

## Overview and Summary

In summary, the risk for developing alcoholism and the resultant negative consequences of alcohol dependence are influenced by a variety of factors in addition to the quantity and frequency of alcohol consumed. Gender, family history, comorbid psychiatric and substance use disorders, and age can impact the development and outcome of alcoholism. This fact significantly complicates the study of alcohol dependence. Ideally, we would construct a straight-forward diagram depicting the interaction of these variables and describing categories into which they might be placed, such as genetic factors, or family factors, or environmental factors. The reality of the complexity of these interactions, however, prohibits readable, meaningful illustration. For example, increasing age generally is associated with decreased risk. However, cohort studies suggest that increasing age might be less protective than it once was. Thus, the interaction of social–cultural issues associated with our current response to healthy aging may reverse the previously reported protective factors of aging. Furthermore, psychiatric comorbidity cannot be comprehensively considered independent of family histories and gender. Although the modulators discussed in this article do not form an “endless” circle, they certainly form a complex system of interconnected factors that eludes illustration.

Despite the difficulties associated with such a complex system, it does identify multiple points of intervention, prevention, and treatment. More specifically, the complexity suggests that there is no single point at which such efforts might be effective. Rather, treatment (broadly defined) may occur at various or multiple intersections and may include behavioral, sociocultural, and pharmacologic interventions. However, to most effectively identify these intersections and treatment modalities, programmatic hypothesis-driven research must be applied.

## Footnotes

1 P300 refers to a positive event-related potential wave recorded via electroencephalography at about 300 to 600 milliseconds. This signal often is used as a measure of cognitive function.

## References

[b1-arh-31-4-389] Agrawal A, Hinrichs AL, Dunn G (2008). Linkage scan for quantitative traits identifies new regions of interest for substance dependence in the Collaborative Study on the Genetics of Alcoholism (COGA) sample. Drug and Alcohol Dependence.

[b2-arh-31-4-389] American Psychiatric Association (APA) (1980). Diagnostic and Statistical Manual of mental Disorders, 3rd Edition (DSM–III).

[b3-arh-31-4-389] APA (1987). Diagnostic and Statistical Manual, 3rd Edition Revised (DSM–III–R).

[b4-arh-31-4-389] APA (1994). Diagnostic and Statistical, Manual, 4th Edition (DSM–IV).

[b5-arh-31-4-389] Ammon E, Schafer C, Hofmann U, Klotz U (1996). Disposition and first-pass metabolism of ethanol in humans: Is it gastric or hepatic and does it depend on gender?. Clinical Pharmacology & Therapeutics.

[b6-arh-31-4-389] Atkinson RM (2002). Alcohol use in later life: Scourge, solace, or safeguard of health?. American Journal of Geriatric Psychiatry.

[b7-arh-31-4-389] Atkinson RM, Misra S, Ryan SC, Turner JA (2003). Referral paths, patient profiles and treatment adherence of older alcoholic men. Journal of Substance Abuse Treatment.

[b8-arh-31-4-389] Aytaclar S, Tarter RE, Kirisci L, Lu S (1999). Association between hyperactivity and executive cognitive functioning in childhood and substance use in early adolescence. Journal of the American Academy of Child and Adolescent Psychiatry.

[b9-arh-31-4-389] Baraona E, Abittan CS, Dohmen K (2001). Gender differences in pharmacokinetics of alcohol. Alcoholism: Clinical and Experimental Research.

[b10-arh-31-4-389] Bartholow BD, Henry EA, Lust SA (2007). Effects of alcohol sensitivity on P3 event-related potential reactivity to alcohol cues. Psychology of Addictive Behaviors.

[b11-arh-31-4-389] Bauer LO, Hesselbrock VM (1999). P300 decrements in teenagers with conduct problems: Implications for substance abuse risk and brain development. Biological Psychiatry.

[b12-arh-31-4-389] Begleiter H, Porjesz B, Bihari B, Kissin B (1984). Event-related brain potentials in boys at risk for alcoholism. Science.

[b13-arh-31-4-389] Blusewicz MJ, Dustman RE, Schenkenberg T, Beck EC (1977). Neuropsychological correlates of chronic alcoholism and aging. Journal of Nervous and Mental Disease.

[b14-arh-31-4-389] Brennan PL, Schutte KK, Moos RH (1999). Reciprocal relations between stressors and drinking behavior: A three-wave panel study of late middle-aged and older women and men. Addiction.

[b15-arh-31-4-389] Carlson SR, Iacono WG, McGue M (2002). P300 amplitude in adolescent twins discordant and concordant for alcohol use disorders. Biological Psychology.

[b16-arh-31-4-389] Carlson SR, Iacono WG, McGue M (2004). P300 amplitude in nonalcoholic adolescent twin pairs who become discordant for alcoholism as adults. Psychophysiology.

[b17-arh-31-4-389] Ceballos NA (2006). Tobacco use, alcohol dependence, and cognitive performance. Journal of General Psychology.

[b18-arh-31-4-389] Ceballos NA, Nixon SJ, Phillips JA, Tivis R (2003). Semantic processing in alcoholics with and without antisocial symptomatology. Journal of Studies on Alcohol.

[b19-arh-31-4-389] Chou SP, Dawson DA (1994). A study of the gender differences in morbidity among individuals diagnosed with alcohol abuse and/or dependence. Journal of Substance Abuse.

[b20-arh-31-4-389] Cloninger CR, Bohman M, Sigvardsson S (1981). Inheritance of alcohol abuse: Cross-fostering analysis of adopted men. Archives of General Psychiatry.

[b21-arh-31-4-389] Collins AC, Marks MJ, Fertig JB, Allen JP (1995). Animal models of alcohol–nicotine interactions. Alcohol and Tobacco: From Basic Science to Clinical Practice. NIAAA Research Monograph No. 30.

[b22-arh-31-4-389] Costa L, Bauer L, Kuperman S (2000). Frontal P300 decrements, alcohol dependence, and antisocial personality disorder. Biological Psychiatry.

[b23-arh-31-4-389] Cotton NS (1979). The familial incidence of alcoholism: A review. Journal of Studies on Alcohol.

[b24-arh-31-4-389] Dawson DA, Goldstein RB, Chou PS (2008). Age at first drink and the first incidence of adult-onset DSM–IV alcohol use disorders. Alcoholism: Clinical and Experimental Research.

[b25-arh-31-4-389] Dettling A, Fischer F, Bohler S (2007). Ethanol elimination rates in men and women in consideration of the calculated liver weight. Alcohol.

[b26-arh-31-4-389] Dick DM, Plunkett J, Hamlin D (2007). Association analyses of the serotonin transporter gene with lifetime depression and alcohol dependence in the Collaborative Study on the Genetics of Alcoholism (COGA) sample. Psychiatric Genetics.

[b27-arh-31-4-389] Diehl A, Croissant B, Batra A (2007). Alcoholism in women: Is it different in onset and outcome compared to men?. European Archives of Psychiatry Clinical Neuroscience.

[b28-arh-31-4-389] DiFranza JR, Guerrera MP (1990). Alcoholism and smoking. Journal of Studies on Alcohol.

[b29-arh-31-4-389] Dixon L, Haas G, Weiden PJ (1991). Drug abuse in schizophrenic patients: Clinical correlates and reasons for use. American Journal of Psychiatry.

[b30-arh-31-4-389] Duncan AE, Scherrer J, Fu Q (2006). Exposure to paternal alcoholism does not predict development of alcohol-use disorders in offspring: Evidence from an offspring-of-twins study. Journal of Studies on Alcohol.

[b31-arh-31-4-389] Durazzo TC, Gazdzinski S, Meyerhoff DJ (2007). The neurobiological and neurocognitive consequences of chronic cigarette smoking in alcohol use disorders. Alcohol and Alcoholism.

[b32-arh-31-4-389] Durazzo TC, Rothlind J, Gazdzinski S (2006). A comparison of neurocognitive function in nonsmoking and chronically smoking short-term abstinent alcoholics. Alcohol.

[b33-arh-31-4-389] Edenberg HJ, Foroud T (2006). The genetics of alcoholism: Identifying specific genes through family studies. Addiction Biology.

[b34-arh-31-4-389] Fabian MS, Parsons OA, Sheldon MD (1984). Effects of gender and alcoholism on verbal and visual-spatial learning. Journal of Nervous and Mental Disease.

[b35-arh-31-4-389] Faeh D, Viswanathan B, Chiolero A (2006). Clustering of smoking, alcohol drinking and cannabis use in adolescents in a rapidly developing country. BMC Public Health.

[b36-arh-31-4-389] Fein G, Chang M (2008). Smaller feedback ERN amplitudes during the BART are associated with a greater family history density of alcohol problems in treatment-naive alcoholics. Drug and Alcohol Dependence.

[b37-arh-31-4-389] Flannery B, Fishbein D, Krupitsky E (2007). Gender differences in neurocognitive functioning among alcohol-dependent Russian patients. Alcoholism: Clinical and Experimental Research.

[b38-arh-31-4-389] Frezza M, di Padova C, Pozzato G (1990). High blood alcohol levels in women: The role of decreased gastric alcohol dehydrogenase activity and first-pass metabolism. New England Journal of Medicine.

[b39-arh-31-4-389] Giancola PR, Martin CS, Tarter RE (1996). Executive cognitive functioning and aggressive behavior in preadolescent boys at high risk for substance abuse/dependence. Journal of Studies on Alcohol.

[b40-arh-31-4-389] Gilman JM, Bjork JM, Hommer DW (2007). Parental alcohol use and brain volumes in early-and late-onset alcoholics. Biological Psychiatry.

[b41-arh-31-4-389] Glenn SW, Errico AL, Parsons OA (1993). The role of antisocial, affective, and childhood behavioral characteristics in alcoholics’ neuropsy-chological performance. Alcoholism: Clinical and Experimental Research.

[b42-arh-31-4-389] Glenn SW, Nixon SJ (1991). Applications of Cloninger’s subtypes in a female alcoholic sample. Alcoholism: Clinical and Experimental Research.

[b43-arh-31-4-389] Glenn SW, Nixon SJ (1996). Investigation of Cloninger’s subtypes in a male alcoholic sample: Applications and implications. Journal of Clinical Psychology.

[b44-arh-31-4-389] Glenn SW, Parsons OA (1989). Alcohol abuse and familial alcoholism: Psychosocial correlates in men and women. Journal of Studies on Alcohol.

[b45-arh-31-4-389] Glenn SW, Parsons OA (1992). Neuropsychological efficiency measures in male and female alcoholics. Journal of Studies on Alcohol.

[b46-arh-31-4-389] Goldstein RB, Dawson DA, Saha TD (2007). Antisocial behavioral syndromes and DSM–IV alcohol use disorders: Results from the National Epidemiologic Survey on Alcohol and Related Conditions. Alcoholism: Clinical and Experimental Research.

[b47-arh-31-4-389] Gomberg ES (1993). Recent developments in alcoholism: Gender issues. Recent Developments in Alcoholism.

[b48-arh-31-4-389] Graff-Radford NR, Heaton RK, Earnest MP, Rudikoff JC (1982). Brain atrophy and neuropsychological impairment in young alcoholics. Journal of Studies on Alcohol.

[b49-arh-31-4-389] Grant BF, Stinson FS, Dawson DA (2004). Prevalence and co-occurrence of substance use disorders and independent mood and anxiety disorders: Results from the National Epidemiologic Survey on Alcohol and Related Conditions. Archives of General Psychiatry.

[b50-arh-31-4-389] Grucza RA, Bierut LJ (2006). Cigarette smoking and the risk for alcohol use disorders among adolescent drinkers. Alcoholism: Clinical and Experimental Research.

[b51-arh-31-4-389] Hasin DS, Stinson FS, Ogburn E, Grant BF (2007). Prevalence, correlates, disability, and comorbidity of DSM–IV alcohol abuse and dependence in the United States: Results from the National Epidemiologic Survey on Alcohol and Related Conditions. Archives of General Psychiatry.

[b52-arh-31-4-389] Heath AC, Bucholz KK, Madden PA (1997). Genetic and environmental contributions to alcohol dependence risk in a national twin sample: Consistency of findings in women and men. Psychological Medicine.

[b53-arh-31-4-389] Heishman SJ (1998). What aspects of human performance are truly enhanced by nicotine?. Addiction.

[b54-arh-31-4-389] Hernandez-Avila CA, Rounsaville BJ, Kranzler HR (2004). Opioid-, cannabis- and alcohol-dependent women show more rapid progression to substance abuse treatment. Drug and Alcohol Dependence.

[b55-arh-31-4-389] Hill SY, Kostelnik B, Holmes B (2007). fMRI BOLD response to the eyes task in offspring from multiplex alcohol dependence families. Alcoholism: Clinical and Experimental Research.

[b56-arh-31-4-389] Hill SY, Muka D, Steinhauer S, Locke J (1995). P300 amplitude decrements in children from families of alcoholic female probands. Biological Psychiatry.

[b57-arh-31-4-389] Hill SY, Shen S (2002). Neurodevelopmental patterns of visual P3b in association with familial risk for alcohol dependence and childhood diagnosis. Biological Psychiatry.

[b58-arh-31-4-389] Hill SY, Shen S, Locke J (1999). Developmental delay in P300 production in children at high risk for developing alcohol-related disorders. Biological Psychiatry.

[b59-arh-31-4-389] Hill SY, Steinhauer SR (1993). Event-related potentials in women at risk for alcoholism. Alcohol.

[b60-arh-31-4-389] Holden KL, McLaughlin EJ, Reilly EL, Overall JE (1988). Accelerated mental aging in alcoholic patients. Journal of Clinical Psychology.

[b61-arh-31-4-389] Hommer D, Momenan R, Kaiser E, Rawlings R (2001). Evidence for a gender-related effect of alcoholism on brain volumes. American Journal of Psychiatry.

[b62-arh-31-4-389] Hommer DW (2003). Male and female sensitivity to alcohol-induced brain damage. Alcohol Research & Health.

[b63-arh-31-4-389] Jacob T, Waterman B, Heath A (2003). Genetic and environmental effects on offspring alcoholism: New insights using an offspring-of-twins design. Archives of General Psychiatry.

[b64-arh-31-4-389] Jang KL, Vernon PA, Livesley WJ (2000). Personality disorder traits, family environment, and alcohol misuse: A multivariate behavioural genetic analysis. Addiction.

[b65-arh-31-4-389] Jang KL, Vernon PA, Livesley WJ (2001). Intra- and extra-familial influences on alcohol and drug misuse: A twin study of gene-environment correlation. Addiction.

[b66-arh-31-4-389] Kaprio J, Pulkkinen L, Rose RJ (2002). Genetic and environmental factors in health-related behaviors: Studies on Finnish twins and twin families. Twin Research.

[b67-arh-31-4-389] Kendler KS, Heath AC, Neale MC (1992). A population-based twin study of alcoholism in women. JAMA: Journal of the American Medical Association.

[b68-arh-31-4-389] Kessler RC, Crum RM, Warner LA (1997). Lifetime co-occurrence of DSM–III–R alcohol abuse and dependence with other psychiatric disorders in the National Comorbidity Survey. Archives of General Psychiatry.

[b69-arh-31-4-389] Krueger RF, Hicks BM, Patrick CJ (2002). Etiologic connections among substance dependence, antisocial behavior, and personality: Modeling the externalizing spectrum. Journal of Abnormal Psychology.

[b70-arh-31-4-389] Mann K, Ackermann K, Croissant B (2005). Neuroimaging of gender differences in alcohol dependence: Are women more vulnerable?. Alcoholism: Clinical and Experimental Research.

[b71-arh-31-4-389] Maurage P, Campanella S, Philippot P (2008). Alcoholism leads to early perceptive alterations, independently of comorbid depressed state: An ERP study. Clinical Neurophysiology.

[b72-arh-31-4-389] McGovern MP, McLellan AT (2008). The status of addiction treatment research with co-occurring substance use and psychiatric disorders. Journal of Substance Abuse Treatment.

[b73-arh-31-4-389] McGue M (1997). A behavioral-genetic perspective on children of alcoholics. Alcohol Health & Research World.

[b74-arh-31-4-389] McGue M (1999). The behavioral genetics of alcoholism. Current Directions in Psychological Science.

[b75-arh-31-4-389] McNamee RL, Dunfee KL, Luna B (2008). Brain activation, response inhibition, and increased risk for substance use disorder. Alcoholism: Clinical and Experimental Research.

[b76-arh-31-4-389] National Projections Program, US Bureau of the Census (2008). National Population Projections Tables and Charts. http://www.census.gov/population/www/projections/tablesandcharts.html.

[b77-arh-31-4-389] Nixon SJ, Glenn SW (1995). Cognitive psychosocial performance and recovery in female alcoholics. Recent Developments in Alcoholism.

[b78-arh-31-4-389] Nixon SJ, Hallford HG, Tivis RD (1996). Neurocognitive function in alcoholic, schizophrenic, and dually diagnosed patients. Psychiatry Research.

[b79-arh-31-4-389] Nixon SJ, Lawton-Craddock A, Tivis R, Ceballos N (2007). Nicotine’s effects on attentional efficiency in alcoholics. Alcoholism: Clinical and Experimental Research.

[b80-arh-31-4-389] Oscar-Berman M, Noronha A, Eckardt M, Warren K (2000). Neuropsychological vulnerabilities in chronic alcoholism. Review of NIAAA’s Neuroscience and Behavioral Research Portfolio. NIAAA Research Monograph No. 34.

[b81-arh-31-4-389] Oscar-Berman M, Marinkovic K (2003). Alcoholism and the brain: An overview. Alcohol Research & Health.

[b82-arh-31-4-389] Oscar-Berman M, Marinkovic K (2007). Alcohol: Effects on neurobehavioral functions and the brain. Neuropsychology Review.

[b83-arh-31-4-389] Parsons OA, Loberg T, Miller Wr, Nathan PE, Marlatt GA (1989). Impairment in sober alcoholics cognitive functioning: The search for determinants. Addictive Behaviors: Prevention and early Intervention.

[b84-arh-31-4-389] Parsons OA (1994). Determinants of cognitive deficits in alcoholics: The search continues. The Clinical Neuropsychologist.

[b85-arh-31-4-389] Pfefferbaum A, Adalsteinsson E, Sullivan EV (2006). Dysmorphology and microstructural degradation of the corpus callosum: Interaction of age and alcoholism. Neurobiology of Aging.

[b86-arh-31-4-389] Pfefferbaum A, Lim KO, Zipursky RB (1992). Brain gray and white matter volume loss accelerates with aging in chronic alcoholics: A quantitative MRI study. Alcoholism: Clinical and Experimental Research.

[b87-arh-31-4-389] Pfefferbaum A, Rosenbloom M, Deshmukh A, Sullivan E (2001). Sex differences in the effects of alcohol on brain structure. American Journal of Psychiatry.

[b88-arh-31-4-389] Pfefferbaum A, Rosenbloom M, Serventi KL, Sullivan EV (2002). Corpus callosum, pons, and cortical white matter in alcoholic women. Alcoholism: Clinical and Experimental Research.

[b89-arh-31-4-389] Pfefferbaum A, Sullivan EV (2002). Microstructural but not macrostructural disruption of white matter in women with chronic alcoholism. NeuroImage.

[b90-arh-31-4-389] Pfefferbaum A, Sullivan EV, Mathalon DH, Lim KO (1997). Frontal lobe volume loss observed with magnetic resonance imaging in older chronic alcoholics. Alcoholism: Clinical and Experimental Research.

[b91-arh-31-4-389] Porjesz B, Rangaswamy M (2007). Neurophysiological endophenotypes, CNS disinhibition, and risk for alcohol dependence and related disorders. ScientificWorldJournal.

[b92-arh-31-4-389] Prescott CA, Kendler KS (1999). Genetic and environmental contributions to alcohol abuse and dependence in a population-based sample of male twins. American Journal of Psychiatry.

[b93-arh-31-4-389] Randall CL, Roberts JS, Del Boca FK (1999). Telescoping of landmark events associated with drinking: A gender comparison. Journal of Studies on Alcohol.

[b94-arh-31-4-389] Rangaswamy M, Jones KA, Porjesz B (2007). Delta and theta oscillations as risk markers in adolescent offspring of alcoholics. International Journal of Psychophysiology.

[b95-arh-31-4-389] Regier DA, Farmer ME, Rae DS (1990). Comorbidity of mental disorders with alcohol and other drug abuse: Results from the Epidemiologic Catchment Area (ECA) Study. JAMA: Journal of the American Medical Association.

[b96-arh-31-4-389] Roache JD, Wang Y, Ait-Daoud N, Johnson BA (2008). Prediction of serotonergic treatment efficacy using age of onset and type A/B typologies of alcoholism. Alcoholism: Clinical and Experimental Research.

[b97-arh-31-4-389] Rodway P, Dienes Z, Schepman A (2000). The effects of cigarette smoking on negative priming. Experimental and Clinical Psychopharmacology.

[b98-arh-31-4-389] Russell MA, Windle M, Searles JS (1990). Prevalence of alcoholism among children of alcoholics. Children of Alcoholics: Critical Perspectives.

[b99-arh-31-4-389] Schutte KK, Brennan PL, Moos RH (1998). Predicting the development of late-life late-onset drinking problems: A 7-year prospective study. Alcoholism: Clinical and Experimental Research.

[b100-arh-31-4-389] Sher KJ, Gotham HJ, Erickson DJ, Wood PK (1996). A prospective, high-risk study of the relationship between tobacco dependence and alcohol use disorders. Alcoholism: Clinical and Experimental Research.

[b101-arh-31-4-389] Sigvardsson S, Bohman M, Cloninger CR (1996). Replication of the Stockholm Adoption Study of alcoholism: Confirmatory cross-fostering analysis. Archives of General Psychiatry.

[b102-arh-31-4-389] Stevens MC, Kaplan RF, Bauer LO (2001). Relationship of cognitive ability to the developmental course of antisocial behavior in substance-dependent patients. Progress in Neuro-Psychopharmacology & Biological Psychiatry.

[b103-arh-31-4-389] Stinson FS, Grant BF, Dawson DA (2005). Comorbidity between DSM–IV alcohol and specific drug use disorders in the United States: Results from the National Epidemiologic Survey on Alcohol and Related Conditions. Drug and Alcohol Dependence.

[b104-arh-31-4-389] Streissguth AP, O’Malley K (2000). Neuropsychiatric implications and long-term consequences of fetal alcohol spectrum disorders. Seminars in Clinical Neuropsychiatry.

[b105-arh-31-4-389] Sullivan EV, Fama R, Rosenbloom MJ, Pfefferbaum A (2002). A profile of neuropsychological deficits in alcoholic women. Neuropsychology.

[b106-arh-31-4-389] Tarter RE (2002). Etiology of adolescent substance abuse: A developmental perspective. American Journal on Addictions.

[b107-arh-31-4-389] Tarter RE, Kirisci L, Mezzich A (2003). Neurobehavioral disinhibition in childhood predicts early age at onset of substance use disorder. American Journal of Psychiatry.

[b108-arh-31-4-389] Thoma P, Wiebel B, Daum I (2007). Response inhibition and cognitive flexibility in schizophrenia with and without comorbid substance use disorder. Schizophrenia Research.

[b109-arh-31-4-389] Thomas SE, Randall PK, Book SW, Randall CL (2008). A complex relationship between co-occurring social anxiety and alcohol use disorders: What effect does treating social anxiety have on drinking?. Alcoholism: Clinical and Experimental Research.

[b110-arh-31-4-389] Uekermann J, Daum I, Schlebusch P (2003). Depression and cognitive functioning in alcoholism. Addiction.

